# Leiomyosarcoma: a rare presentation as multifocal lesion

**DOI:** 10.1259/bjrcr.20190117

**Published:** 2020-05-11

**Authors:** Mohanad Kareem Aftan, Afra Alfalahi, Ethar Alzeena, Usama albastaki, Yamina Houcinat, Khalid Mahmoud

**Affiliations:** 1 Rashid Hospital, Dubai Health Authority, Dubai, United Arab Emirates; 2 Jadara Radiology Center, Amman, Jordan

## Abstract

Leiomyosarcoma is a rare type of connective tissue cancer, accounting for 5–10% of all soft tissue sarcomas. We present a case of leiomyosarcoma as unusual multifocal presentation. Retroperitoneal, mediastinal, pulmonary, uterine and bony regions were all involved at the time of presentation. The liver was normal without detected lesions.

## Case presentation

**Figure 1.**
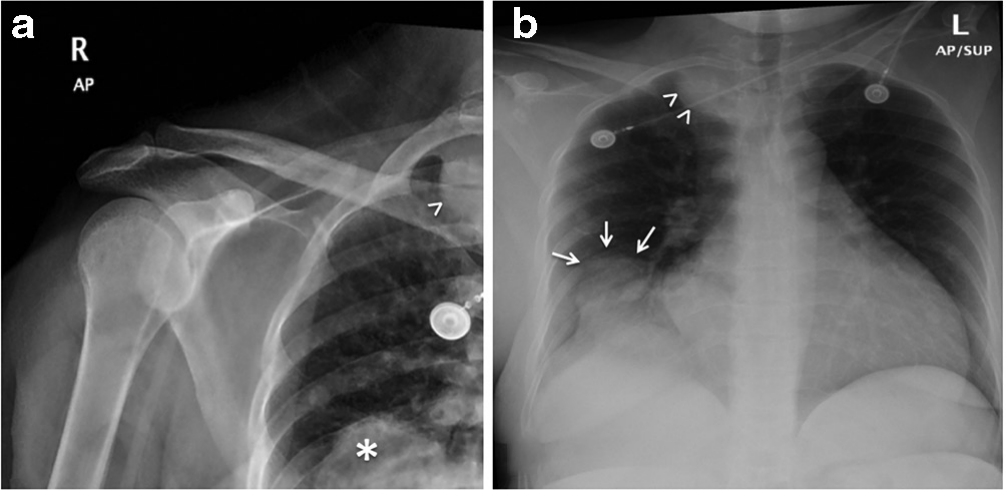
(A) AP view of the right shows a soft tissue opacity projecting over the bottom right corner of the film (white asterisk), another mediastinally based mass (arrow head). (B) AP view portable chest X-ray confirms the presennce of masss in the lower zone of right lung (white arrows) and the above mentioned mediastinal mass (arrow heads). AP, anteroposterior.

A 50-year-old lady presented to the emergency department with a history of right shoulder pain for 4 days. Right shoulder X-ray was done and showed a mediastinal mass at the edge of the film (Figure 1A). The patient had other associated symptoms, including fatigue, dyspnea on exertion, dizziness and weight loss of 10 kg over 1 year. She noticed a growing abdominal mass over the past few months but didn't seek any medical advice. She denied any melena, hematuria, hematemesis or heavy menstruation. For this reason, multiple imaging studies were performed including the following: chest radiograph, chest, abdomen and pelvis CT scan with intravenous contrast and lumber spine MRI with contrast.

### Investigations

**Figure 2.**
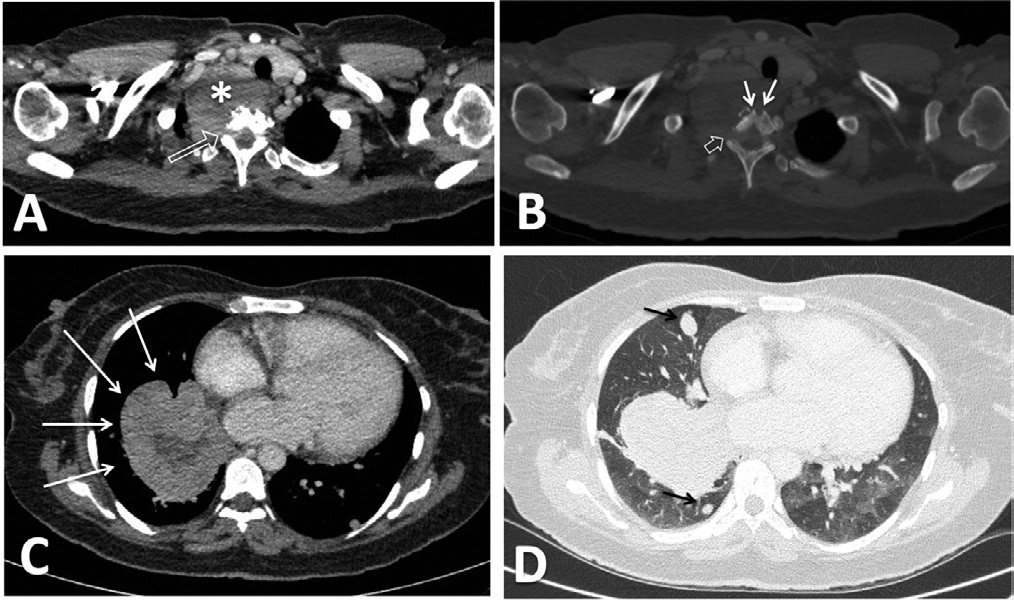
CT chest with i.v. contrast. (A) Mediastinal window at the level of lung apex shows multilobulated heterogeneously enhancing soft tissue mass with multiple hypo-enhancing/necrotic areas (white asterisk), It directly extends through the right nerve root exit foramen to the spinal canal at the same level (long white open arrow). (B) Bone window at the same level shows posterior extension to the adjacent D1 vertebral body causing infiltrative lytic bony changes (white arrows) and directly extending through the right nerve root exit foramen to the spinal canal at the same level (white open arrow). (C) soft tissue window at a lower level shows another multilobulated soft tissue mass with central necrotic areas involving the medial, anterior and basal aspects of the right lower lobe. It abuts but does not invade the adjacent pericardium (long white arrows). (D) Lung window shows multiple nodules (short black arrows) likely metastatic in nature.

**Figure 3.**
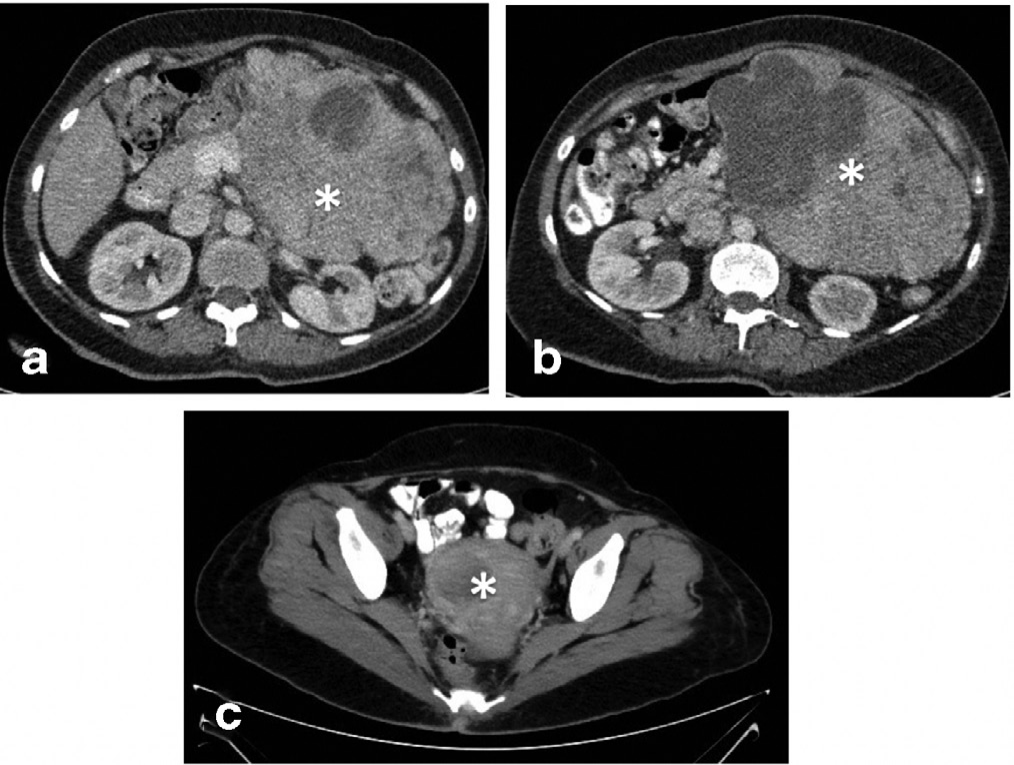
Abdomen and pelvis CT scan with IV contrast axial cuts. (A, B) Multilobulated soft tissue mass with necrotic center (White asterisk). Its epicenter at the left upper aspect of the peritoneum. It displaces but not invades the adjacent bowel loops, vascular structures and pancreas. (C) At a lower level shows another lesion with similar radiological characteristics in the uterus (white asterisk).

Hematological evaluation revealed an elevated erythrocyte sedimentation rate with severe anemia. Chest CT scan with intravenous contrast showed a multilobulated heterogeneously enhancing mediastinal mass with multiple hypoenhancing/necrotic areas (Figure 2A&B). It caused destruction and infiltration of the adjacent D1 vertebral body and directly extended through the right nerve root exit foramen to the spinal canal at the same level. Multilobulated lung parenchymal soft tissue mass with central necrotic areas involving medial, anterior and basal aspects of the right lower lobe (Figure 2C). It abutted, but did not invade, the adjacent pericardium. Lung window showed multiple nodules likely metastatic in nature (Figure 2D). Abdomen and pelvis CT scan with intravenous contrast showed a retroperitoneal mass (Figure 3A&B) with similar characteristics to the aforementioned right lung mass. The mass located with its epicenter at the left upper aspect of the peritoneum. The adjacent bowel loops, vascular structures and pancreas were displaced with no local invasion noted. A fourth mass was found in the uterus with similar characteristics of other primary tumors (Figure 3C).

**Figure 4.**
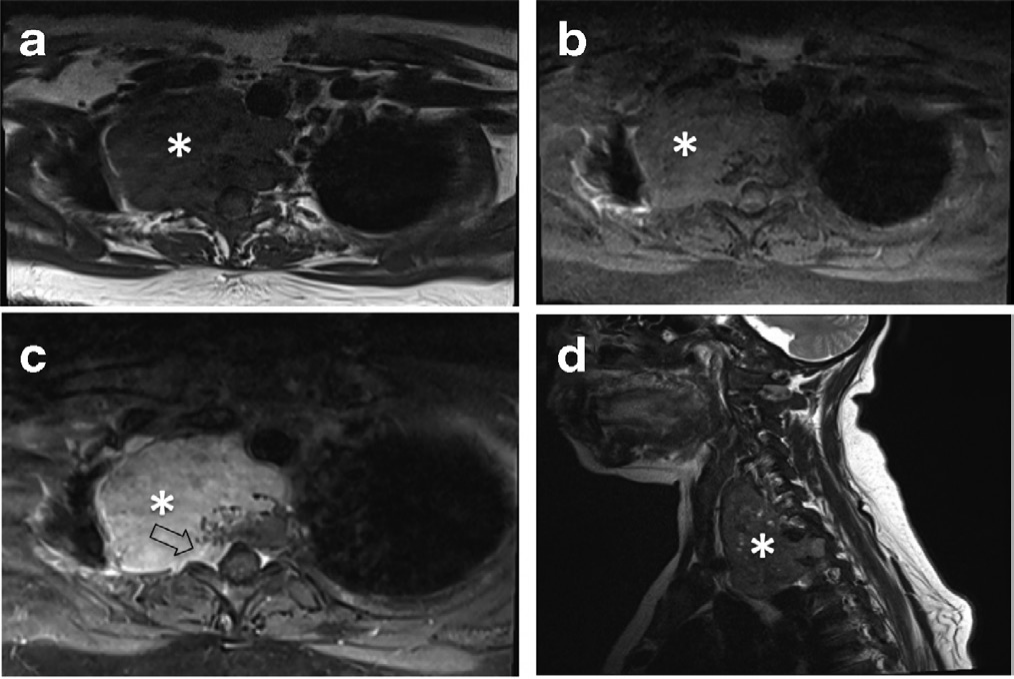
MRI. The mass is labeled with white asterisk. (A) Axial T1 sequence shows heterogeneously iso-Intense signal relative to muscle signal. (B) Axial STIR sequence shows heterogeneously mildly hyperintense mass relative to muscle signal. STIR, short tau inversion recovery. (C) Axial T1 sequence post contrast shows heterogeneous hyperenhancement with involvement of D1 vertebral body and extension into the spinal canal through the right neural foramen (open black arrow). (D) Sagittal T2 sequence shows its cranial extension to cricoid cartilage level.

Cervical spine MRI with intravenous contrast showed the superior mediastinal mass with T1 sequence heterogeneously isointense signal, short tau inversion recovery sequence heterogeneously mildly hyperintense signal relative to muscle signal and T1 post-contrast sequence heterogeneous hyperenhancement with involvement of D1 vertebral body and extension into the spinal canal through the right neural foramen (Figure 4A). Sagittal T2 sequence showed its cranial extension to the level of cricoid cartilage (Figure 4D).

**Figure 5.**
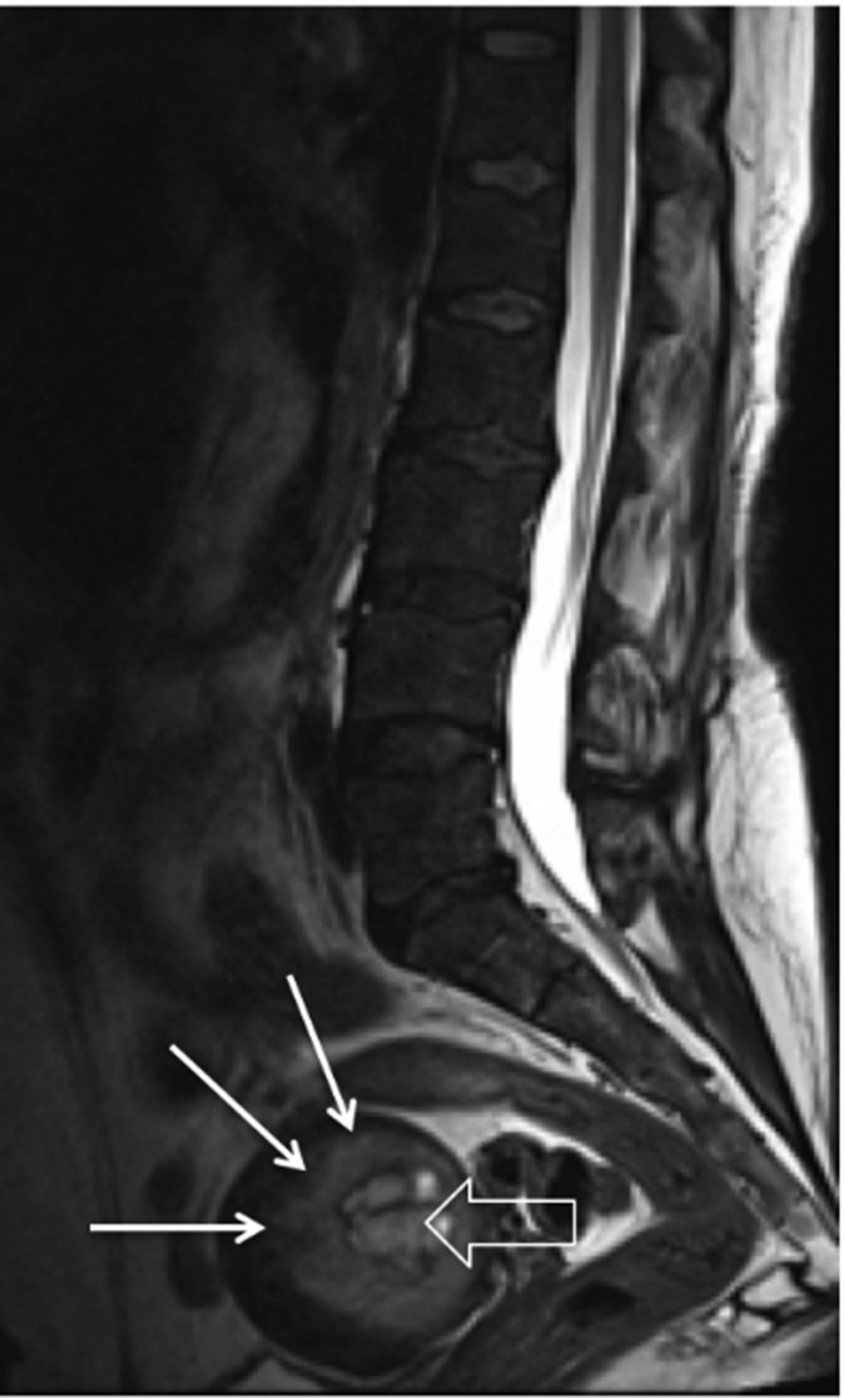
MR lumbar spine T2 sequence sagittal cut shows similar soft tissue lesion within the uterus (long white arrows) with central area of necrosis (open white arrow).

Lumber spine MRI with intravenous contrast showed a uterine mass with irregular margins and central cystic/hemorrhagic areas (Figure 5). The diffuse bone marrow signal intensity reduction is due to chronic anemia.

**Figure 6.**
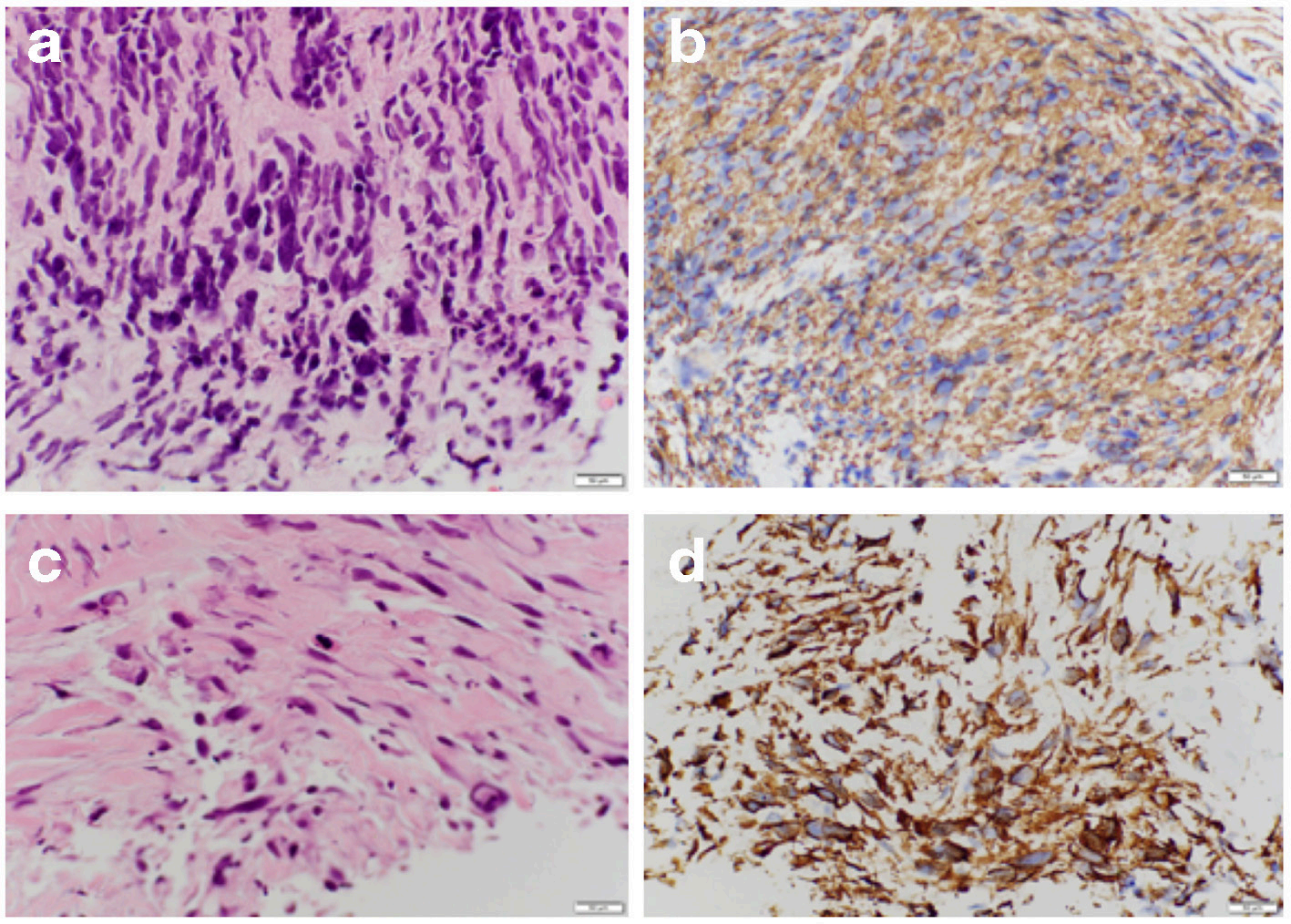
Histopathology of ultrasound guided biopsy of the retroperitoneal mass. (A) High power photomicrograph shows spindle cells, hyperchromatic nuclei, anesochariosis, pleomorphism and atypia. (B) SMA stain shows positive smooth muscle tumor. (C) Mitosis. (D) smooth muscle marker caldesmon. Other markers for other spindle cell tumors where negative. MDM2 test for de-differentiated liposarcoma is negative. Tests for lymphoma, nerve sheath tumor, solitary fibrous tumor is negative. SMA, smooth muscle actin.

Histopathology of ultrasound-guided biopsy of the retroperitoneal mass confirmed the diagnosis of high grade leiomyosarcoma (Figure 6).

## Discussion

Soft tissue sarcomas comprise 0.7% of adult malignancies.^1^ Leiomyosarcoma is a malignant neoplasm that shows smooth muscle differentiation.^2^ It is predominantly observed in elderly females, typically females between ages of 50 and 70 s, however, males may also be affected. The vast majority of these lesions are located in the abdominal cavity and pelvic regions.

**Mediastinal leiomyosarcoma** is a rare neoplasm, accounting for about 1.4% of soft tissue sarcomas and for about 11% of primary mediastinal sarcomas.^3^ Mediastinal leiomyosarcomas are usually large masses showing heterogenous contrast enhancement due to the presence of hemorrhagic and necrotic components. When present in the mediastinum, leiomyosarcomas typically present clinically as a result of local mass effect. Lung parenchymal primary leiomyosarcomas usually present as a well-defined lesion with smooth or lobulated margins. They may show a necrotic center as well.^4^ Leiomyosarcoma is one of the more common histologic subtypes of sarcoma to occur in the lung.^5,6^ **Retroperitoneal leiomyosarcoma** is the second most common sarcoma to affect the retroperitoneum, comprising about 28% of cases.^7^ It has three patterns of growth. Entirely intraluminal leiomyosarcoma is the least common (5%). Leiomyosarcoma growth with both intra- and extravascular components is accounting for 33% of cases. In over 60% of cases, entirely extravascular retroperitoneal sarcoma represents the most common growth pattern. Metastases from extravascular retroperitoneal leiomyosarcoma are seen in follow-up studies in most of the cases and in only about 9% of patients at time of presentation.^8^ The majority of retroperitoneal leiomyosarcomas are typicaly seen arising from the perirenal or posterior pararenal spaces.^8^ Lungs are the most common site for retroperitoneal leiomyosarcoma metastasis, accounting for 65%, followed by peritoneum (53%), liver (53%), muscle (41%), bones (35%) and lymph nodes (35%).^8^

**Uterine leiomyosarcomas** account for only 1–2% of uterine malignancies and occur mainly after menopause. The great majority arise *de novo*, but rarely (in 0.2% of cases) it may result from a sarcomatous transformation in a benign leiomyoma.^9^ Pre-operative distinction between benign leiomyomas and malignant leiomyosarcomas is very difficult (if not impossible) based solely on clinical features and remains a challenge for clinicians.^10,11^ Because of increased cellular density, these lesions may display restricted diffusion. The combination of diffusion-weighted imaging and T2 signal intensity lead to increase MRI accuracy in differentiating between benign and malignant or uncertain tumors affecting the myometrium. Thomassin-Naggara et al^12^ concluded that diffusion-weighted imaging sequence has to be the first measure to be used to help reduce the wrong diagnosis of uterine sarcomas as benign leiomyomas.

**Extremities and trunk leiomyosarcoma** usually present as a painful single mass. It appears as indistinct mass that is rarely calcified or locally invades adjacent bony structures on radiography. On MRI scan, these lesions appear as heterogenous soft tissue masses with central necrosis. The survival rates of patients with extremities and trunk leiomyosarcoma in 5 and 10 year are 64 and 46%, retrospectively, with a metastatic rate of about 34%.^13^ Soft tissue sarcoma of extremities and trunk rarely metastasize to the liver as compared with visceral and retroperitoneal soft tissue sarcoma, which usually show hepatic metastasis.^14^ The majority of extremities and trunk soft tissue sarcoma metastasis go to the lung (88%) while lymph nodes and other soft tissues metastases from these lesions account for approximately 12%.^15^

In our patient, although there were supradiaphragmatic, infradiaphragmatic lesions and multiple lung metastases, the liver was spared with no signs of metastasis. In addition, the behavior and the radiological appearance of the lesions in the mediastinum, lung, retroperitoneum and uterus were almost similar to a solitary leiomyosarcoma affecting each area alone.

## Differential diagnosis

### Lymphoma: (Both Hodgkin & non-Hodgkin):

Mass-like conglomeration of lymph nodes, more strongly resembling leiomyosarcoma.^16^ Most often it appears as well defined and homogeneous with mild contrast enhancement.^17^ Commonly, it displaces structures, including aorta from the spine and typically does not invade the major vascular structures.^18^ Moreover, it may be associated with other places of extra nodal lymphoma, mainly in gastrointestinal tract involvement.^16^ It rarely presents with calcification or necrosis without prior treatment. The associated clinical history of fever, night sweating, weight loss and the associated increase in the serum LDH level may raise the suspicion of lymphoma over leiomyosarcoma.^19^

### Lymph node metastasis

Generally, testicular carcinoma, prostate adenocarcinoma, renal cell carcinoma, and cervical carcinoma commonly spread to retroperitoneal lymph nodes.^20,21^ History of primary tumor and tumor markers elevation suggest distant metastasis over leiomyosarcoma.^21,22^

### Soft tissue sarcoma

High grade liposarcoma and leiomyosarcoma are the most common soft tissue sarcoma affecting the retroperitoneum. Radiologically, it is difficult to differentiate between liposarcoma and leiomyosarcoma if there is no macroscopic fat. Approximately, 30% of liposarcoma present with calcification, which is an uncommon finding in leiomyosarcoma.^23^

### Neurogenic tumor

Neurogenic tumors include nerve sheath tumors, tumors of the sympathetic ganglia and extra-adrenal paragangliomas. Clinical and imaging features include young patient age, paraspinal location and calcification. Absence of vascular involvement may help distinguish nerve sheath tumors and tumors of the sympathetic ganglia from a leiomyosarcoma. Paraganglioma mostly located at the organ of Zuckerkandl.

## Learning points

1. Retroperitoneal leiomyosarcomas usually appear as large heterogenous lesions due to intralesional areas of necrosis, hemorrhage or cystic areas. These lesions rarely show calcifications without osteosarcomatous differentiation. Classically, they arise in the perirenal or posterior pararenal spaces.
2. Lung parenchymal primary leiomyosarcoma usually present as well-defined lesion with smooth or lobulated margins.
3. Liver metastases from extremities and trunk leiomyosarcoma is rare in comparison with retroperitoneal and visceral leiomyosarcoma. Only approximately 9% of cases show metastases at time of presentation. The majority of cases show metastases on follow-up studies.
4. Because of increased cellular density, these lesions may display restricted diffusion. This is a very important characteristic used to differentiate uterine leiomyoma from leiomyosarcoma.
